# Renoprotective Effect of Lactoferrin against Chromium-Induced Acute Kidney Injury in Rats: Involvement of IL-18 and IGF-1 Inhibition

**DOI:** 10.1371/journal.pone.0151486

**Published:** 2016-03-18

**Authors:** Rehab Hegazy, Abeer Salama, Dina Mansour, Azza Hassan

**Affiliations:** 1 Pharmacology Department, Medical Division, National Research Centre, Giza, Egypt; 2 Pathology Department, Faculty of Veterinary Medicine, Cairo University, Giza, Egypt; National Institutes of Health, UNITED STATES

## Abstract

Hexavalent chromium (CrVI) is a heavy metal widely used in more than 50 industries. Nephrotoxicity is a major adverse effect of chromium poisoning. The present study investigated the potential renoprotective effect of lactoferrin (Lf) against potassium dichromate (PDC)-induced acute kidney injury (AKI) in rats. Beside, because previous studies suggest that interlukin-18 (IL-18) and insulin-like growth factor-1 (IGF-1) play important roles in promoting kidney damage, the present work aimed to evaluate the involvement of these two cytokines in PDC model of AKI and in the potential renoprotective effect of lactoferrin. Adult male albino Wistar rats were pretreated with Lf (200mg/kg/day, p.o.) or (300mg/kg/day, p.o.); the doses that are usually used in the experiment studies, for 14 days followed by a single dose of PDC (15mg/kg, s.c.). PDC caused significant increase in serum urea, creatinine, and total protein levels. This was accompanied with decreased renal glutathione content, and increased renal malondialdehyde, IL-18, IL-4, nuclear factor kappa B (NFκB), IGF-1, and the phosphorylated form of forkhead box protein O1 (FoxO1) levels. Moreover, normal expression IFN-γ mRNA and enhanced expression of TNF-α mRNA was demonstrated in renal tissues. Histopathological investigations provoked deleterious changes in the renal tissues. Tubular epithelial hyperplasia and apoptosis were demonstrated immunohistochemically by positive proliferating cell nuclear antigen (PCNA), Bax, and Caspase-3 expression, respectively. Pretreatment of rats with Lf in both doses significantly corrected all previously mentioned PDC-induced changes with no significant difference between both doses. In conclusion, the findings of the present study demonstrated the involvement of oxidative stress, inflammatory reactions, tubular hyperplasia and apoptosis in PDC-induced AKI. It suggested a role of IL-18 through stimulation of IL-4-induced inflammatory pathway, and IGF-1 through triggering FoxO1-induced cell proliferation. Moreover, the study revealed that Lf protected the kidney against Cr-induced AKI in rats and significantly showed antioxidant, anti-inflammatory, and anti-proliferative properties with down-regulation of IL-18 and IGF-1.

## Introduction

Chromium (Cr) is a heavy metal with several valence states; the most common of which is the hexavalent (CrVI). Hexavalent chromium (CrVI) is widely used in more than 50 industries, for stainless steel manufacturing, leather tanning, chrome plating, welding and wood processing [1,2]. Occupational and environmental exposure to CrVI-containing compounds are known to be toxic and carcinogenic to human beings and diverse animals [3]. Nephrotoxicity is a major adverse effect of Cr poisoning due to the fact that the main known route for chromium excretion is through the kidney with a resultant increase in its chromium content and subsequently, nephropathy [4]. The toxic manifestations of Cr are attributed primarily to oxidative stress [5,6] leading to serious damage to the vital organs [7,8].

The role of inflammation in acute kidney injury (AKI) has been increasingly appreciated with involvement of leukocytes, adhesion molecules, chemokines, and cytokines. Interleukin-18 (IL-18) is a pro-inflammatory cytokine that is produced by proximal renal tubular cells and has been proven to play an important role in AKI and is a potential mediator of tubular damage [9,10]. On the other hand, insulin-like growth factor-1 (IGF-1) is a multifunctional hormone that has pleiotropic effects on cellular proliferation, apoptosis, hypertrophy, and differentiation [11]. It has been found to play a pathogenic role in proliferation of renal tubular epithelial cells and renal cyst formation [11–13]. However, the involvement of these molecules in Cr-induced AKI has not been investigated yet.

Lactoferrin (Lf) is a natural iron-binding glycoprotein that is found predominantly in milk and also in other mucosal secretions and bodily fluids [14,15]. Lf has multi-pharmacological properties that are mediated through specific receptors present on the surface of many cells [16,17]. It has been reported to possess anti-bacterial, anti-fungal, anti-parasitic, anti-viral [16–19], anti-inflammatory [20], and antioxidant [21] properties. Moreover, It has been found that Lf enhances apoptosis [22] and it shows anti-carcinogenic [23–25] and immunoregulatory properties[26].

In a screening for Lf expression in various organs, high levels of Lf-mRNA and protein were detected in the kidneys. This indicated that Lf is produced by kidneys and that Lf may have important functions in both innate immunity of this organ as well as in the antioxidant and other defence systems protecting kidneys against any other non-microbial injuries, such as ischemia-reperfusion and inflammation [27]. In the current study, the protective effect of two doses of Lf, that are usually used in the experiment studies, has been assessed against a PDC-induced AKI rat model. We also investigated the role of IL-18 and IGF-1 in the pathogenesis of PDC-induced AKI, and the effect of co-administration of Lf on their renal levels.

## Materials and Methods

### Animals

Adult male albino Wistar rats, weighing 200–250g, were obtained from the animal house colony of National Research Centre (NRC) (Giza, Egypt). The animals were maintained at a controlled temperature of 24 ± 1°C with a 12–12 h light-dark cycle (light cycle, 07:00–19:00). They were allowed free access to water and standard chow *ad libitum*. The animals were treated according to the national and international ethics guidelines stated by the ethics committee of NRC and all procedures and experiments were performed according to the protocol approved by it, and the earliest scientifically justified endpoint was used in this study to prevent pain or distress in the experimental animals.

### Drugs and chemicals

PDC was purchased from Sigma Aldrich Chemical Co. (USA) and Lf was purchased from Radiance Nutritional Company (New Zealand).

### Experimental Design

Thirty-six rats were randomly allocated into six groups (n = 6). The first group received saline and served as a normal group. Rats of groups 2 and 3 were treated with Lf (200 mg/kg/day, p.o.) [22] and Lf (300 mg/kg/day, p.o.) [28], respectively, for 14 days and served as control groups. In the other 3 groups, acute nephrotoxicity was induced by single injections of PDC (15 mg/kg, s.c.) [29] following treatment with saline, Lf (200 mg/kg/day, p.o.) or Lf (300 mg/kg/day, p.o.), respectively, for 14 days.

### Serum biochemical analysis

Twenty four hours following the last PDC injection, blood samples were withdrawn from rats of all groups via retro-orbital vein under light ether anesthesia [30]. Serum was used for estimation of serum urea, creatinine and total protein levels, using specific diagnostic kits (Biodiagnostic, Egypt).

### Renal tissue biochemical analysis

Immediately after blood sampling, animals were sacrificed by cervical dislocation under ether anaesthesia. No animal died prior to this experimental endpoint. The two kidneys from each rat were immediately dissected out, and rinsed with PBS to remove excess blood. Weighed parts from both kidneys were homogenized (MPW-120 homogenizer, Med instruments, Poland) in PBS to obtain 20% homogenate that stored overnight at ≤ –20°C. After two freeze-thaw cycles were performed to break the cell membranes, the homogenates were centrifuged for 5 minutes at 5000 x g using a cooling centrifuge (Sigma and laborzentrifugen, 2k15, Germany). The supernatant was removed immediately and assayed for reduced glutathione (GSH) and lipid peroxides, measured as malondialdehyde (MDA), contents using (Biodiagnostic, Egypt) kits. Moreover, renal contents of IL-18, IL-4, nuclear factor kappa B (NFκB), IGF-1, and the phosphorylated form of forkhead box protein O1 (FoxO1) were also assessed using specific diagnostic kits, (R&D Systems, USA), (RayBiotech, USA), (Glory Science, USA), (Antagene, USA), and (Cloud-Clone Crop, USA), respectively.

### Real-time reverse transcriptase-polymerase chain reaction (RT-PCR) assay

The number of the mRNA copies of interferon gamma (IFN-γ) and tumor necrosis factor alpha (TNF-α) was assessed by quantitative RT-PCR in RNA extracts from renal tissue homogenates from rats of all groups. Total RNA was extracted using the RNeasy Mini Kit (QIAGEN, Germany) as described in the manufacturer’s protocol. RT-PCR assays to specifically quantify rat IFN-γ and TNF-α mRNA were performed using a specific diagnostic kits (SNP Biotechnology R&D Ltd., USA) and a detection system (Step one Plus^™^ Real time device, Applied Biosystems, USA), according to the manufacturer’s instructions.

### Histopathological examination

Other parts of kidneys from all groups were fixed in 10% neutral buffered formalin for 72 h at least, washed, dehydrated, and embedded in paraffin. Sections of 5μm thickness were stained with Hematoxylin and Eosin (H&E) [31]for routine histopathological examination. Five renal sections per group were examined. Ten random high microscopic fields(x40) per section were examined for assessment of the histopathological lesions using binocular Olympus CX31 microscope. Grades of tubular damage were scored according to the method described before [32,33], with some modifications. Grade 1 (very slight) describes swelling of renal tubular epithelium; Grade 2 (mild): granular degeneration of renal tubular epithelium; Grade 3 (moderate): granular and/or vacuolar degeneration of renal tubular epithelium with presence of few intracytoplasmic hyaline droplets; and Grade 4 (severe): tubular necrosis with presence of intraluminal renal casts as well as interstitial inflammation.

### Immunohistochemical analysis

Demonstration of proliferating cell nuclear antigen (PCNA) immunoreactivity in renal sections of normal and treated rats was performed according to the method described by Eldridge and Goldsworthy [34]. Tissue sections were deparaffinized and incubated with a monoclonal antibody to PCNA (Dako Corp, Carpenteria, CA). It was then incubated with biotin-conjugated secondary antibody (Vactastain ABC peroxidase kit, Vector Laboratories). The immunoreaction was visualized using the chromagen 3, 3-diaminobenzidine tetrahydrochloride (DAB, Sigma Chemical Co.). The positively PCNA immunostained cells were counted in three random high microscopic fields per each group.

Demonstration of Bax and activated Caspase-3 immunostaining in kidney sections of normal and treated rats, as apoptotic markers, was performed according to the method described by Ibrahim et al.,2015 [35]. Rabbit anti-caspase-3 (diluted to 1:1000, Abcam, Ltd., USA) and Bax (1:200, Abcam, Ltd.,USA) were used as biotinylated primary antibodies. Colour intensity of positive immune-reactive cells was determined in 10 random low microscopic field (X10) using Image analyzer (Leica Qwin 500, Cambride, Engalnd). The image was transformed into a grey image [a grid of pixels each representing the intensity or brightness at that point by a range of numbers, typically from 0 (black) to 255 (white)]. A greyscale image is a colour mode that displays image using 256 shades of grey, referred to as 8-bit greyscale image. Each colour was defined as a value between 0 and 255, where 0 is the darkest (black) and 255 is the lightest (white).

### Statistical Analysis

All the values are presented as means ± standard error of the means (SE). Comparisons between different groups were carried out using one-way analysis of variance (ANOVA) followed by Tukey HSD test for multiple comparisons [36]. Graphpad Prism software, version 5 (Inc., San Diego, USA) was used to carry out these statistical tests. The difference was considered significant when *p* < 0.05.

## Results

### Renal function markers

**Fig 1:**
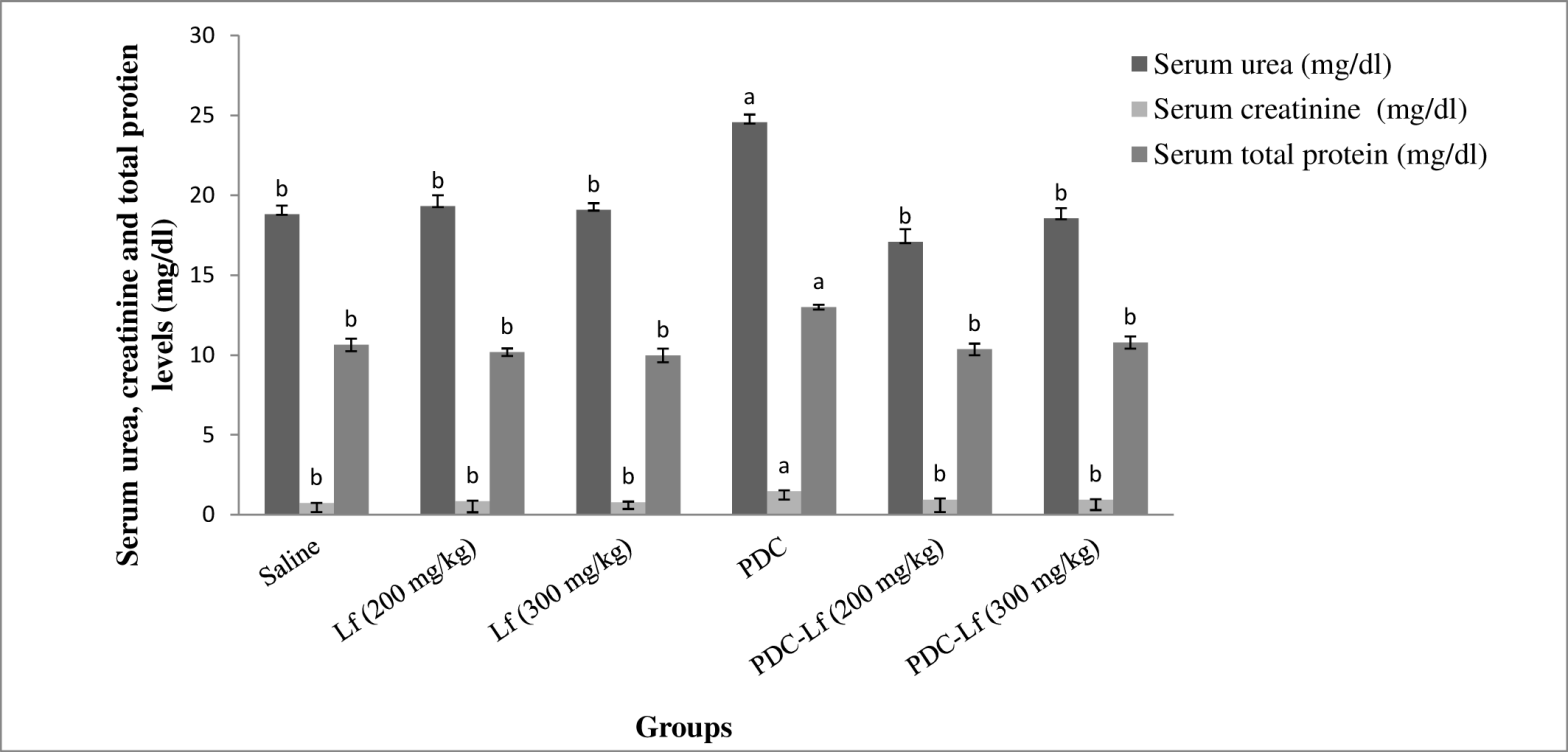
Serum levels of urea, creatinine and total protein. Saline, rats treated with Saline; Lf, rats treated with lactoferrin; PDC, rats treated with potassium dichromate; PDC-Lf, rats treated with potassium dichromate and lactoferrin. ^a^ Significantly different from normal group at *p* < 0.05. ^b^ Significantly different from PDC at *p* <0.05.

Administration of Lf (200 mg and 300 mg/kg) showed no effect on the normal levels of renal function biomarkers of rats. However, a significant elevation in serum urea, creatinine and total protein levels was observed in PDC-treated rats compared with those of normal group. Pretreatment of rats with both doses of Lf restored the normal levels of serum urea and creatinine elevated by PDC, with no significant difference between them (Fig 1).

### Oxidative stress markers

**Fig 2:**
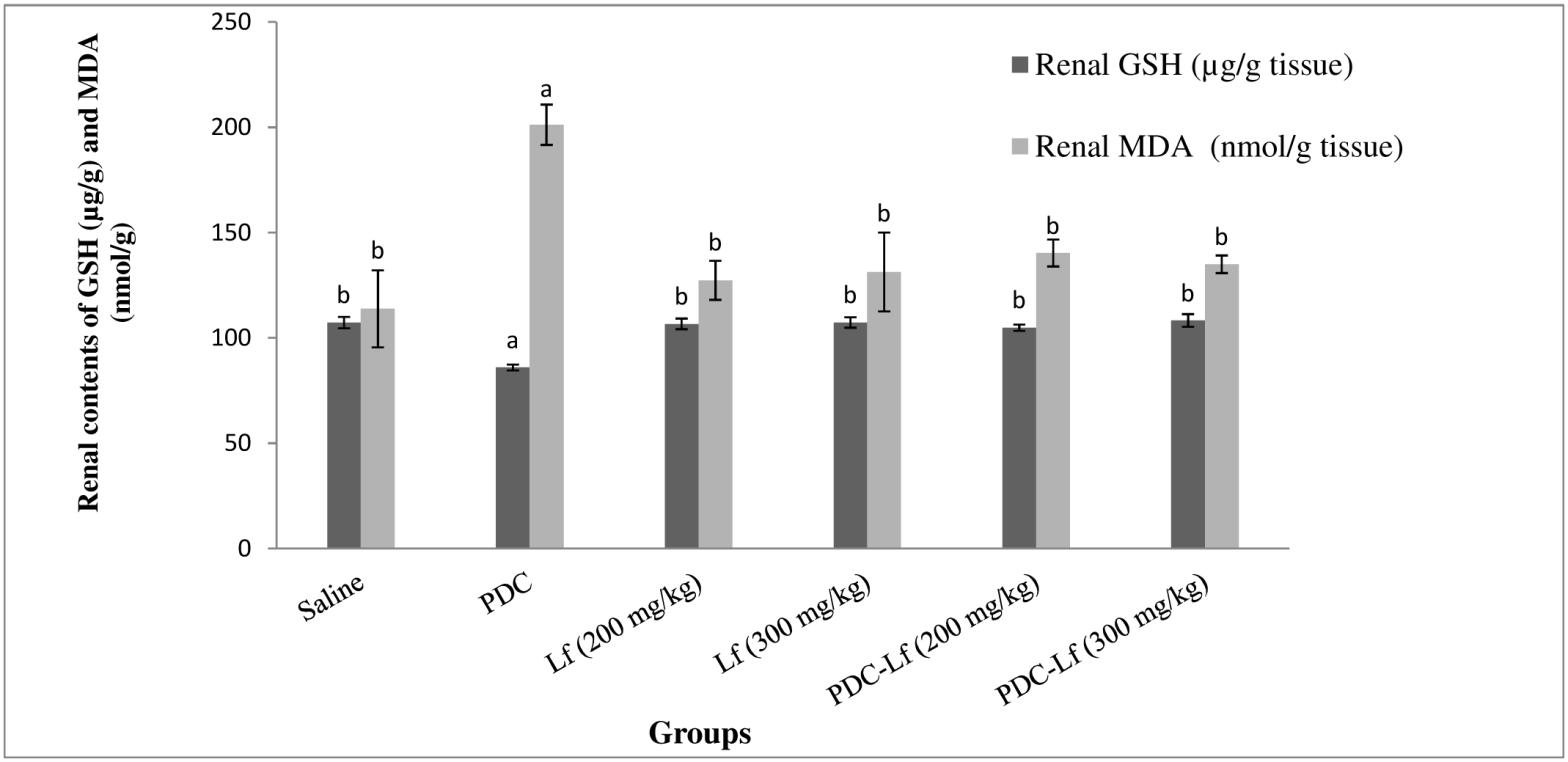
Renal contents of oxidative stress markers; GSH and MDA. Saline, rats treated with Saline; Lf, rats treated with lactoferrin; PDC, rats treated with potassium dichromate; PDC-Lf, rats treated with potassium dichromate and lactoferrin. ^a^ Significantly different from normal group at *p* < 0.05. ^b^ Significantly different from PDC at *p* <0.05.

Normal levels of Oxidative stress biomarkers were observed in control groups treated with Lf (200 mg and 300 mg/kg). On the other hand, renal GSH content was significantly reduced following PDC-administration, and a significant elevation of renal MDA content was detected. Treatment of rats with Lf, in its two doses, for 14 days prior to PDC injection significantly retrieved the altered levels of GSH and MDA (Fig 2).

### Renal contents of IL-18, IL-4, NFκB, IGF-1, and FoxO1

**Fig 3:**
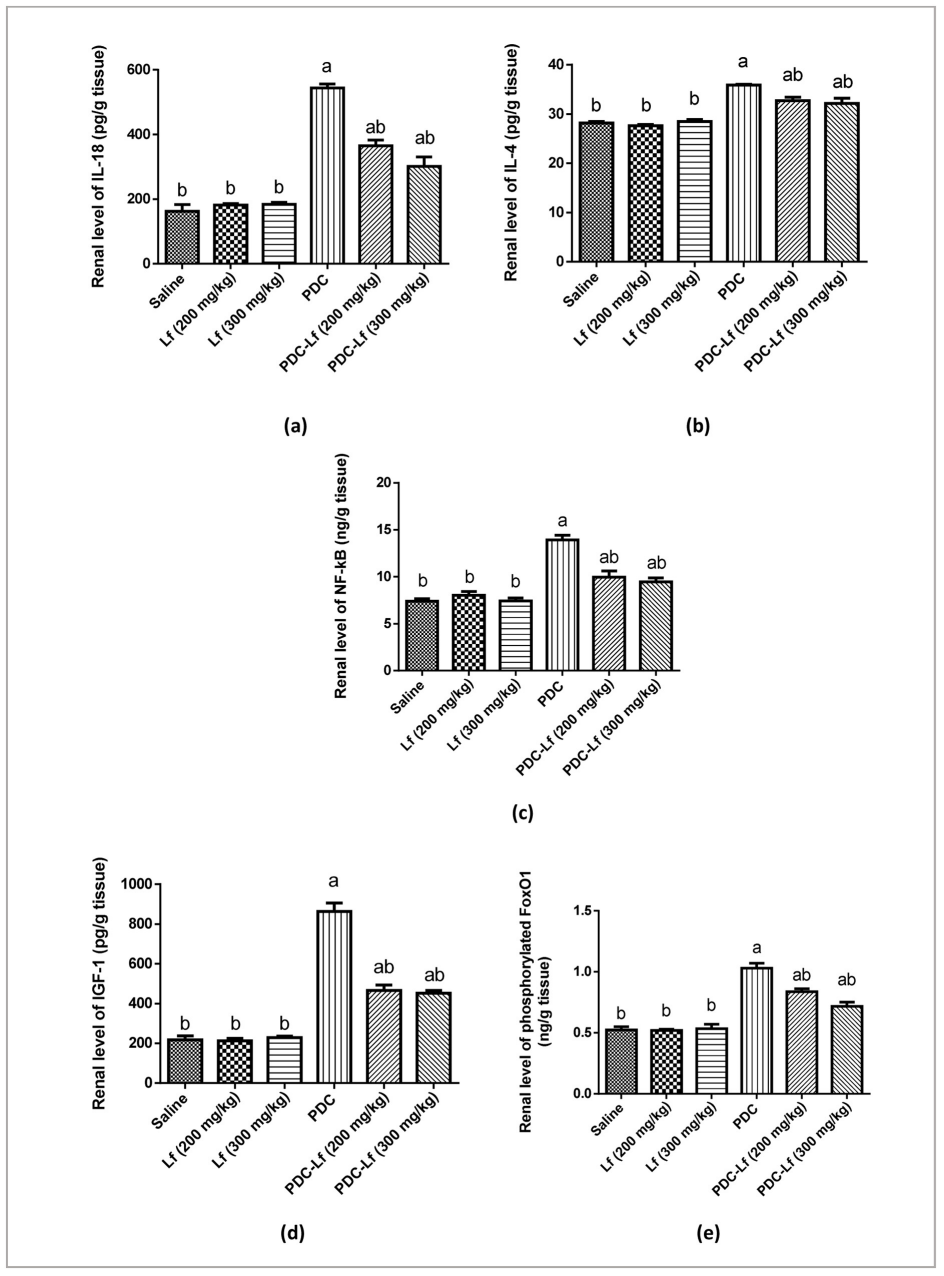
Renal contents of IL-18, IL-4, NFκB, IGF-1, and FoxO1. Saline, rats treated with Saline; Lf, rats treated with lactoferrin; PDC, rats treated with potassium dichromate; PDC-Lf, rats treated with potassium dichromate and lactoferrin. ^a^ Significantly different from normal group at *p* < 0.05. ^b^ Significantly different from PDC at *p* <0.05.

Administration of Lf (200 mg and 300 mg/kg) showed no effect on the normal levels of the measured cytokines in the renal tissues of rats. Induction of AKI in rats with PDC increased the normal kidney contents of IL-18, IL-4, NFκB, IGF-1, and phosphorylated FoxO1. Pretreatment of rats with Lf significantly decreased PDC-induced elevation renal contents of IL-18 and IGF-1, with no significant difference between the effects of both doses of Lf (Fig 3).

### RT-PCR quantification of IFN-γ and TNF-α mRNA in the kidney

**Fig 4:**
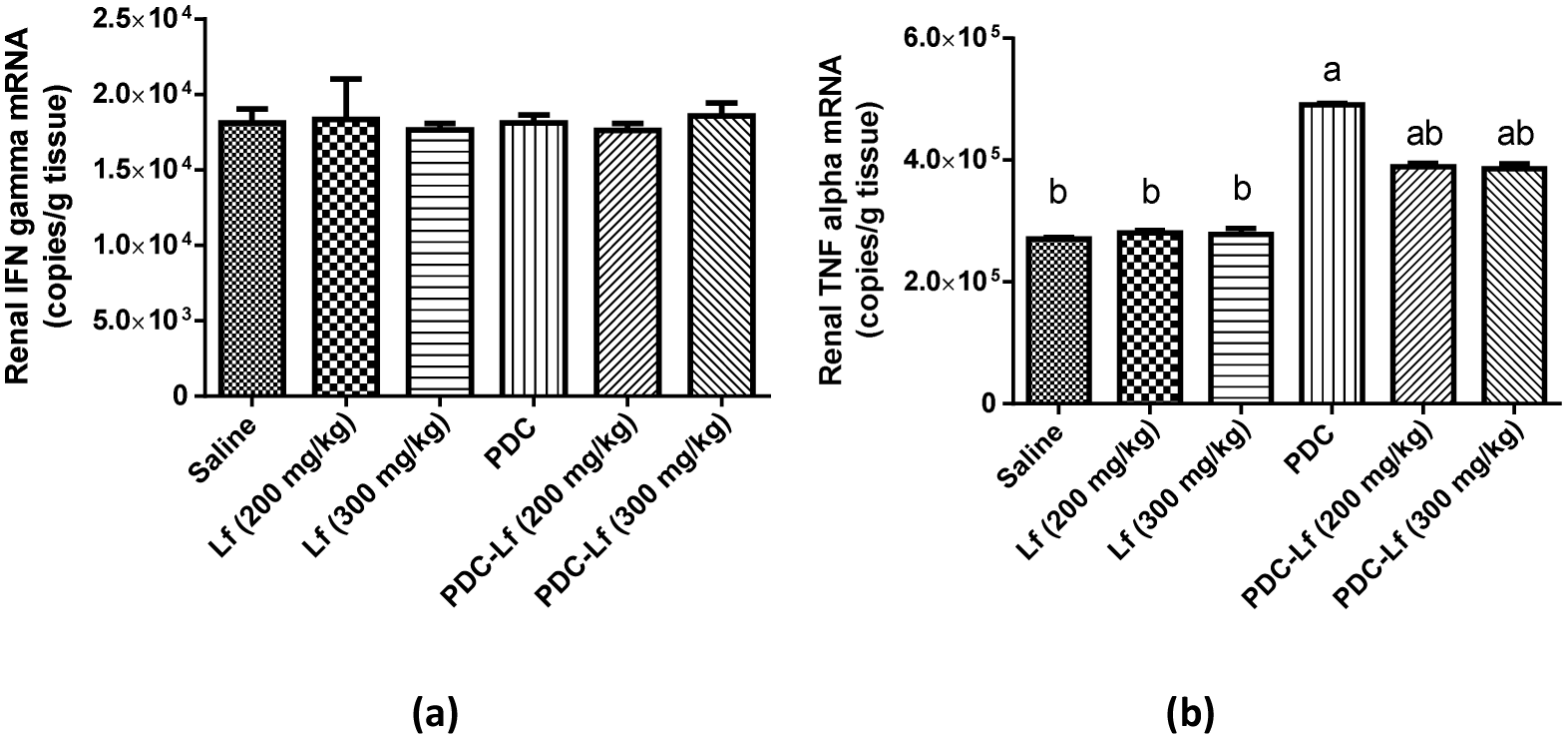
RT-PCR analysis of expression of IFN-γ and TNF-α mRNA in the renal tissues. Saline, rats treated with Saline; Lf, rats treated with lactoferrin; PDC, rats treated with potassium dichromate; PDC-Lf, rats treated with potassium dichromate and lactoferrin. ^a^ Significantly different from normal group at P < 0.05. ^b^ Significantly different from PDC at P <0.05.

No significant difference was detected between the mean levels of mRNA copies for IFN-γ in the groups studied in this experiment (Fig 4a). On the other hand, the mean levels of mRNA copies for TNF-α in Lf control groups were not significantly different when compared with the normal group, while, PDC-treated and PDC-Lf treated groups demonstrated significantly higher mean TNF-α gene expression than the normal. However, in PDC-Lf treated groups, the mean TNF-α mRNA copy numbers were markedly lower than in PDC-treated group (Fig 4b)

### Histopathology

**Fig 5:**
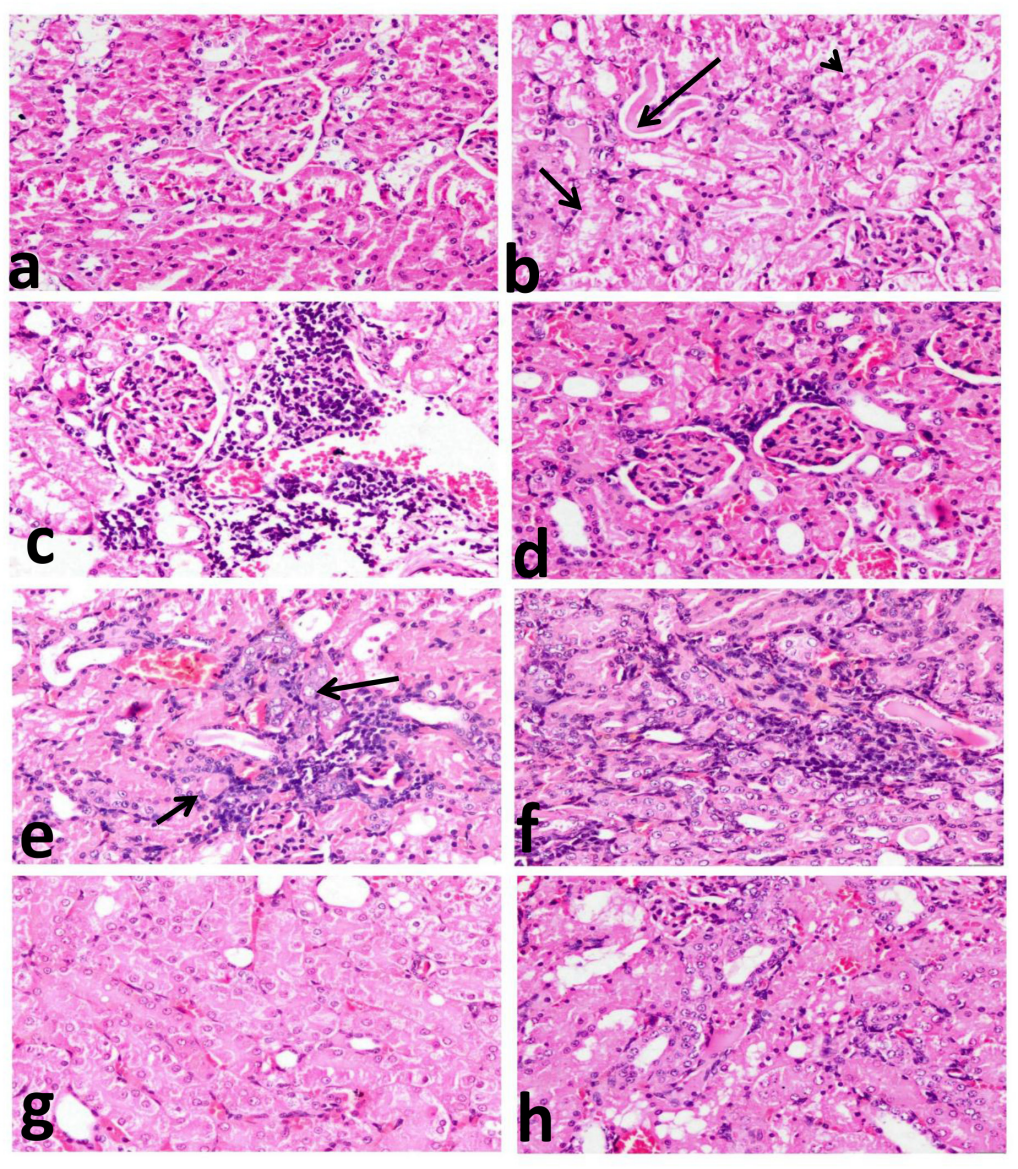
Histopathological investigation of renal tissues. kidney sections of: **normal rats (a)**; showing normal glomeruli and renal tubules, **PDC- treated rats (b, c, d, e and f)** showing: (b); coaggulative necrosis of their epithelial lining with pyknotic nuclei and intracytoplasmic aggregation of hyaline droplets (short arrows) as well as intraluminal renal cast (long arrow) in addition to apoptosis (arrow head), (c); congestion of interstitial blood vessel and glomerular capillary tuft associated with intense focal renal interstitial proliferating mononuclear cell infiltration, (d); thickening of glomerular basement membrane with focal periglomerular lymphocytic cell infiltration, (e); tubular epithelial hyperplasia with karyomegally, nuclear atypia (long arrow) and binucleation (short arrows), (f) tubular epithelial hyperplasia associated with fibroblastic proliferation and mononuclear cell infiltration; **PDC-Lf (200 mg/kg) treated rats** (g); showing mild tubular damage with granular degeneration of their epithelail lining, and **PDC-Lf (300 mg/kg) treated rats** (h); showing focal necrosis of epithelial lining associated with uniformly arranged regenerative renal tubules.(H&E, X400).

The kidney sections of the rats of normal and Lf-treated control groups showed normal histological structures with normal glomeruli and renal tubules (Fig 5a), whereas variable and severe histopathological alterations (Grade 4 tubular damage, 3.60±1.14) (Table 1) in the form of degenerative, inflammatory, and hyperplastic lesions, were demonstrated in PDC-treated group. All proximal convoluted tubules revealed severe necrobioteic changes varying from diffuse vacuolar degeneration to coaggulative necrosis of their epithelial lining with pyknotic nuclei and intracytoplasmic as well as intraluminal aggregation of hyaline droplets and renal cast (Fig 5b). Congestion of interstitial blood vessel and glomerular capillary tuft associated with intense focal renal interstitial proliferating mononuclear cell infiltration (Fig 5c) was frequently demonstrated. Glomeruli showed thickening of glomerular basement membrane with focal periglomerular lymphocytic cell infiltration (Fig 5d). One of the most characteristic histopathological alterations demonstrated in this group was foci of tubular epithelial hyperplasia with karyomegally, nuclear atypia, and binucleation (Fig 5e) associated with fibroblastic proliferation and mononuclear cell infiltration (Fig 5f). These alterations were markedly alleviated in PDC-Lf (200 mg/kg) group (Grade 2 tubular damage, 1.60±0.51) (Table 1) as renal tubules showed mild tubular damage with granular degeneration of their epithelail lining (Fig 5g) and very mild inflammatory cell infiltrates in addition to few regenerative renal tubules. Kidneys of PDC-Lf (300 mg/kg) group showed marked improvements compared to PDC-treated one, with minimal inflammatory reaction, absence of nuclear atypia, and moderate tubular damage (Grade 3 tubular damage, 2.40±0.76) (Table 1) that is demonstrated in only one examined section, characterized by focal necrosis of epithelial lining associated with uniformly arranged regenerative renal tubules (Fig 5h).

**Table 1:** Histopathological and immunohistochemical findings.

Groups	Parameters			
	Tubular Damage (Grade)	PCNA (Count/High microscopic field)	Bax (Greyscale /Low microscopic field)	Caspase-3 (Greyscale/Low microscopic field)
**Saline**	1.90^b^ ± 0.60	______	______	______
**PDC**	3.60^a^ ± 1.14	149^a^ ± 86.41	190.60^a^ ± 10.74	114.67^a^ ± 45.79
**PDC-Lf (200 mg/kg)**	1.60^b^ ± 0.51	59.33^a^^b^ ± 34.26	193.20^a^^b^ ± 13.01	182.71^a^^b^ ± 27.04
**PDC-Lf (300 mg/kg)**	2.40^b^ ± 0.76	91.33^a^^b^ ± 52.73	207.60 ^a^^b^ ±8.64	169.99^a^^b^ ± 29.51

Saline, rats treated with Saline; PDC, rats treated with potassium dichromate; PDC-Lf, rats treated with potassium dichromate and lactoferrin; PCNA, proliferating cell nuclear antigen expression in renal sections counted in three random high microscopic field per group.

Data are presented as mean ± SE.

^a^ Significantly different from normal group at P < 0.05

^b^ Significantly different from PDC at P <0.05.

### Immunohistochemistry

**Fig 6:**
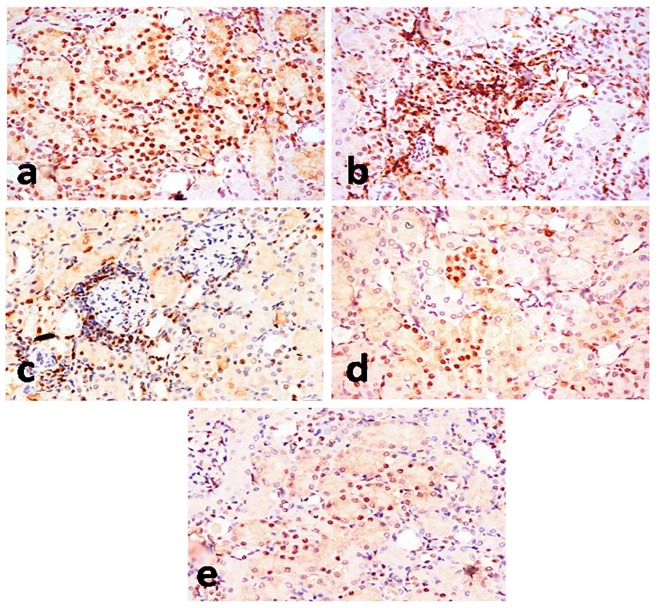
PCNA-immunohistochemical staining of kidney section of rats. Kidney sections of **PDC-treated group (a, b, c)** showing significant increase of PCNA-positive renal tubular cells **(a)**, PCNA-positive proliferating mononuclear cells in interstitial tissue **(b)**, and PCNA-positive proliferating mononuclear cells in the periglomerular area **(c)**. Kidney sections of **PDC-Lf (200 mg/kg)** treated rats **(d)** showing decrease of PCNA-positive renal tubular cells, and **PDC-Lf (300 mg/kg)** treated rats (e) decrease of PCNA-positive renal tubular cells. (Immunohistochemical staining of PCNA, X400).

In the normal control group and Lf control groups, PCNA-positive cells were not found in the kidney sections. Abundant PCNA-positive renal tubular cells (Fig 6a) and proliferating mononuclear cells in renal interstitial tissue and periglomerular area (Fig 6b and 6c) were demonstrated in PDC-treated group (149.67±86.41) compared to Lf-pretreated groups (59.33±34.26 & 91.33±52.73, for Lf 200 mg/kg and 300 mg/kg, respectively) (Fig 6d and 6e) with no significant difference between both doses of Lf (Table 1).

**Fig 7:**
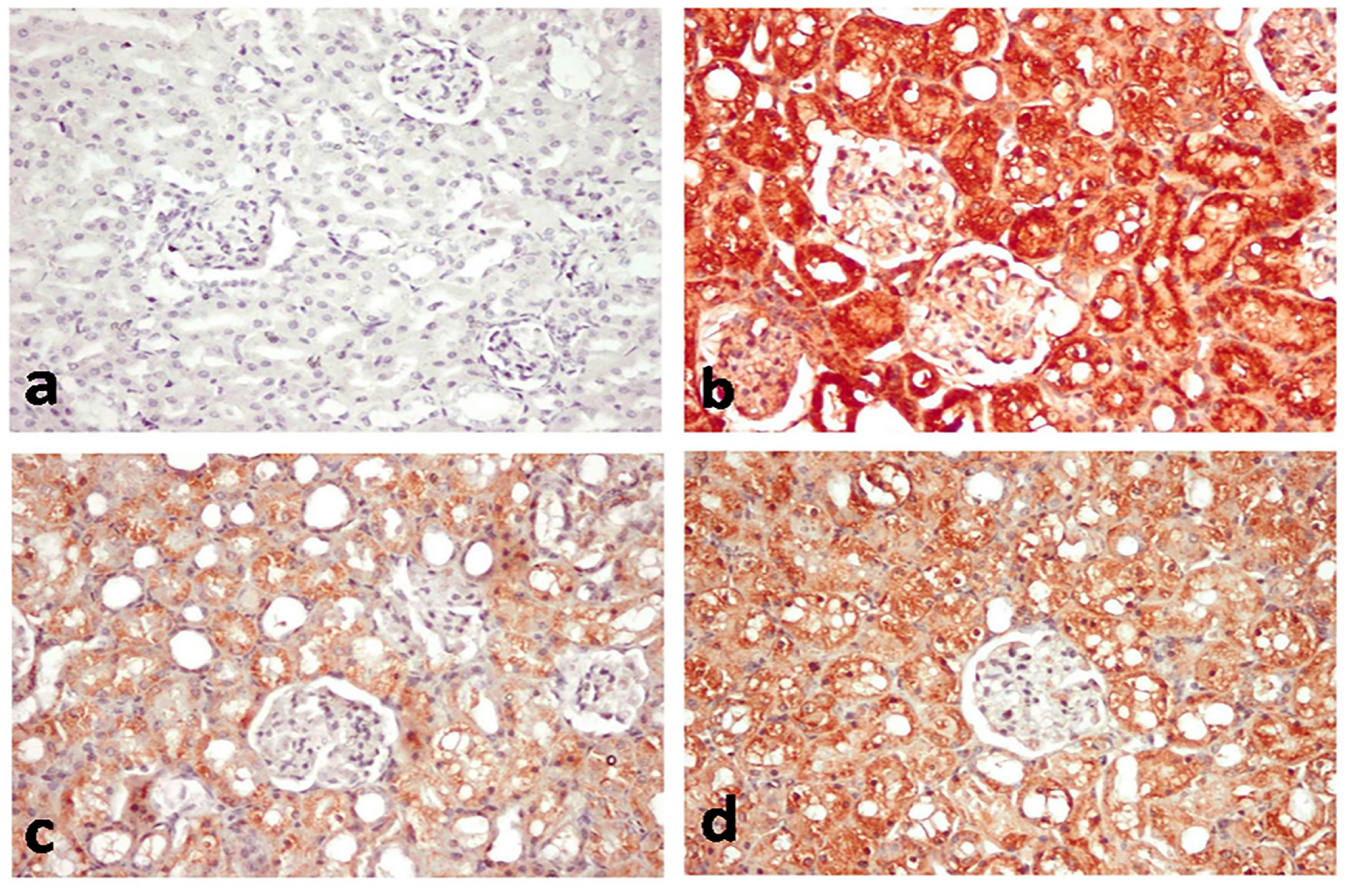
Bax-immunohistochemical staining of kidney section of rats. Kidney sections of **normal rats (a)** showing no immune-reactive cells, **PDC-treated group (b)** showing diffuse intensely stained glomerular and renal tubular immune-reactive cells, **PDC-Lf (200 mg/kg) treated rats (c)** showing faintly stained immune-reactive cells, and **PDC-Lf (300 mg/kg) treated rats (d)** showing moderately stained immune-reactive cells. (immunohistochemical staining of Bax, X400).

**Fig 8:**
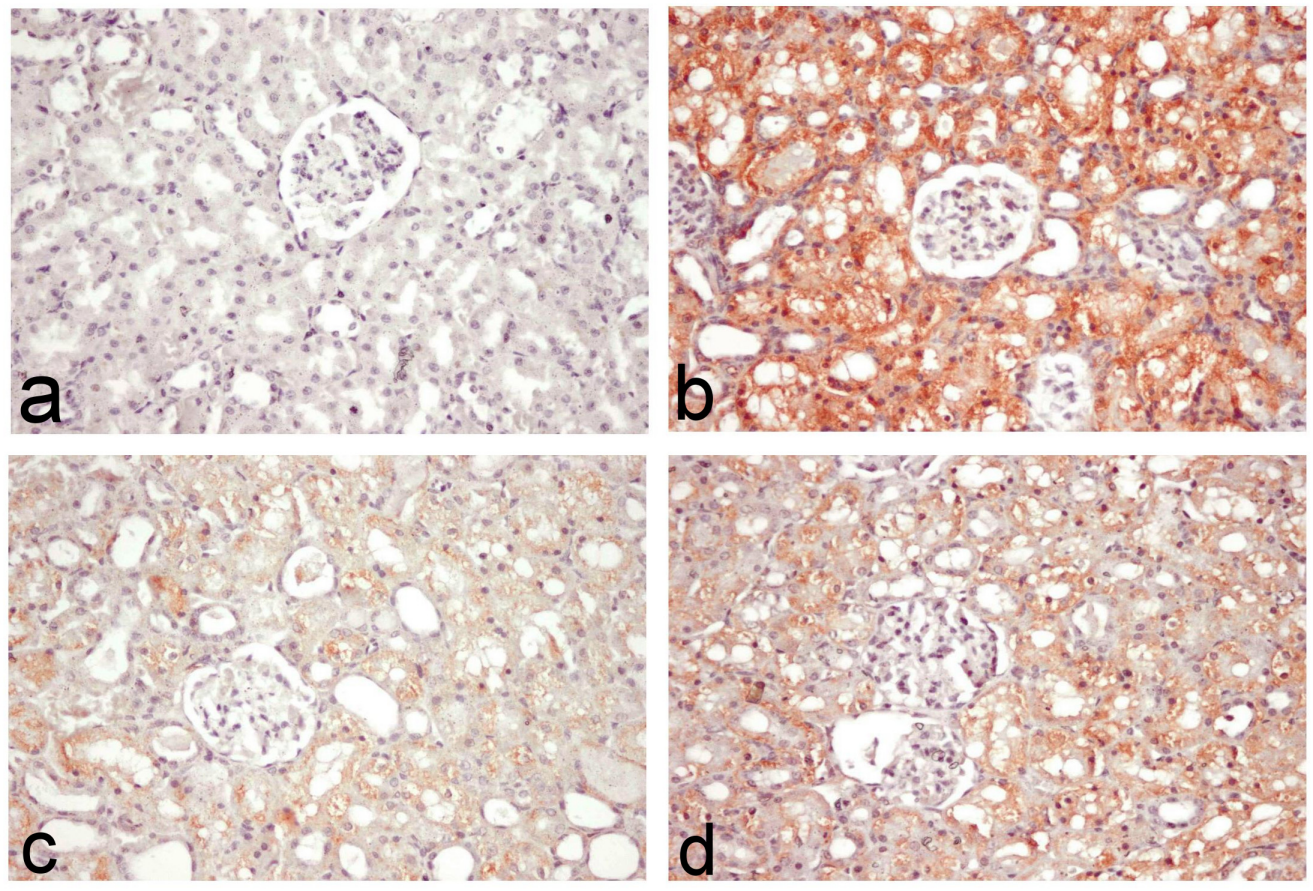
Caspase-3 immunohistochemical staining of kidney section of rats. Kidney sections of **normal rats (a)** showing no immune-reactive cells, **PDC-treated group (b)** showing Diffuse intensely stained immune-reactive cells, **PDC-Lf (200 mg/kg) treated rats (c)** showing few number of faintly stained immune-reactive cells, and **PDC-Lf (300 mg/kg) treated rats** showing decreased numbers of immune-reactive cells. (immunohistochemical staining of Caspase-3, X400).

Figs 7 and 8 outlined the results of immunohistochemical assessment of Bax and Caspase-3 immunoreactive cells in the kidney tissues. No immune reaction was demonstrated in normal and Lf control groups (Figs 7a and 8a). Diffuse intensely stained glomerular and renal tubular epithelial cells were demonstrated in the PDC-treated group (Figs 7b and 8b), with grey level scaled as 114.67±45.79 for Bax and 190.6±10.74 for caspase-3 (Table 1). On the other hand, colour intensity was greatly reduced in PDC-Lf (200 mg/kg)-treated group (Figs 7c and 8c), with grey level scaled as 182.71±27.04 for Bax and 193.2±13.01 for caspase-3 (Table 1). In PDC-Lf (300 mg/kg)-treated group, colour intensity was also reduced compared to PDC-treated group (Figs 7d and 8d), with grey level scaled as 169.99±29.51 for Bax and 207.6±8.64 for caspase-3 (Table 1).

## Discussion

Injection of a single dose of PDC, in the present study, induced AKI in rats as evidenced by the significant alteration of the serum levels of kidney function biomarkers and confirmed with the severe histopathological changes, particularly in the proximal convoluted tubules of PDC-treated group. These results are denoting the nephrotoxic effect of PDC, and are in accordance with those reported by other studies [29,37–39]. The toxic effect of PDC has been attributed to the massive reactive oxygen species (ROS) production, resulting from the reduction of hexavalent chromium to trivalent one, which induces damage of cellular components including proteins, lipids, and DNA [40]. The Kidney is the target organ of systemically absorbed chromate, and nephrotoxicity or complete renal shut down could be the primary cause of death in acute chrome exposure [4]. The tubular damage and nephrotoxic effect of chromate resulted from its accumulation in vacuoles inside the proximal tubular cells that lead to slow excretion and remaining of Cr in the kidney for a long time [41].

In the current study, increased level of MDA (a marker of lipid peroxidation) and decreased level GSH in the kidney tissues of PDC-treated rats confirm those explanations and are in accordance with many other studies [29,37].

Pretreatment of rat with Lf markedly protected the kidney against PDC-induced AKI and oxidative stress, as evidenced by restoration of serum levels of kidney function biomarkers and histopathological changes as well as prevention of MDA accumulation, and maintenance of normal GSH renal contents. However, no significant difference was observed between the effects of the two doses of Lf that are usually used in the experimental studies; 200 and 300 mg/kg. In line to the findings of the present study, Kimoto *et*. *al*. [28] demonstrated protective effect of Lf against cisplatin-induced nephrotoxicity in rats. In addition, many studies reported the antioxidant effect of Lf [22,42,43], and mentioned that the binding of Lf to the cells limited the process of membrane lipid peroxidation, because Lf is not entirely saturated and is capable to scavenge free iron radicals that are cytotoxic activators of the lipid peroxidation and oxidative stress and subsequently suppress free radical-mediated damage [44].

Induction of AKI in rats with PDC in the present study resulted in significant elevation of kidney IL-18 content. IL-18 is a pro-inflammatory cytokine that is induced and cleaved in the proximal tubule [9]. It has been proven to play an important role in AKI, and to be a potential mediator of tubular damage [9,10]. Urinary IL-18 is considered as an early biomarker for the diagnosis of AKI, and on multivariate analysis, urine IL-18 levels predicted the development of AKI 24 hours before the rise in serum creatinine, with an AUC of 0.73 [45]. Corresponding to the result of the current study, high levels of IL-18 were previously detected in the kidney tissues of AKI-animal models. Significant up-regulation of IL-18 expression was reported within mice kidneys at 24 h after ischemia/reperfusion [10] as well as in those with cisplatin-induced AKI [46]. IL-18 is known to promote inflammation and immunity through its key cellular targets including macrophages, T cells, and NK cells, which leads to inflammation and subsequent kidney injury [47].

IL-18 was initially identified as a factor that enhances IFN-γ production by T helper 1 (Th1) cells together with IL-12; however, IL-18 stimulates a Th2 response when it acts alone or in synergy with IL-2 [48]. The Th2 effect of IL-18 includes production of IL-4 by CD4+T cells, basophils, and mast cells [49]. In the present study, the elevated level of IL-18 in PDC-treated group is accompanied by elevated level of IL-4, while, there was no significant difference observed in IFN-γ mRNA expression. These results suggest that in the current PDC-AKI model, IL-18 acted through stimulation of Th2 response and production of IL-4.

IL-4 is the most important determinant of immunoglobulin (Ig E) production that directly elicit allergic inflammatory responses [50]. Therefore, the factors that induce IL-4 production are intimately associated with the pathogenesis of allergic inflammatory reactions. In these reactions, large amount of TNF-α is produced, which activate the endothelial cells, causing increased expression of adhesion molecules that increase the influx of inflammatory leukocytes and lymphocytes into tissues. In agreement with this explanation, the current study demonstrated a marked increase in TNF-α mRNA level in the renal tissues of PDC-treated rats. The histopathological and immunohistochemical findings also confirmed the biochemical results in which inflammatory response was observed in PDC-treated group with increased number of proliferating lymphoid cells. Correspondingly, Beaver *et*.*al*. [51] reported induction of acute inflammatory reaction with neutrophils, macrophages, lymphocytic cell infiltration induced by Cr.

Pretreatment of rats with both doses of Lf significantly decreased the renal content of IL-18, IL-4, and TNF-α compared to the PDC-treated group. These observations confirm and extend previous studies reporting that Lf possesses potent anti-inflammatory effects that inhibit pro-inflammatory cytokines such as TNF-α, interferon-gamma, and inflammatory cytokines such as IL-1β, IL-2, IL-4, IL-6 and IL-10 [20,52–54]. Cumberbatch *et*.*al*. [55] demonstrated reduction of epidermal Langerhans’ cell migration away from the epidermis, which is dependent upon the local availability of IL-1b, IL-18 and TNF-α, with Lf. In contrast, other studies reported enhanced expression of IL-18 induced by Lf in the small intestine of tumor-bearing mice [56,57]. However, increased and decreased production of pro-inflammatory cytokines by Lf according to the requirement has been studied and examined [58–60]. Lf receptors are present on various immune cells, and their capability to bind with Lf shows the specificity and potential ability of Lf to modulate the response of innate and adaptive systems [61]. For that, Lf is known to play a vital role as a mediator of systemic inflammatory response syndrome by allowing the controlled regulation of inflammation without any pathological damage [61,62].

In this study, the anti-inflammatory effect of Lf was confirmed by the histopathological findings that showed marked improvements of renal tissues with mild granular and/or vacuolar degeneration of the lining epithelium of renal tubules with no evidence of interstitial inflammatory cell infiltrate in rats treated by both low and high doses of Lf. Also, the inhibition of peripheral blood mononuclear cells proliferation was confirmed in histopathological and immunohistochemical examinations in this study.

In the present study, PDC-induced AKI was associated with elevated level of renal NF-κB. ROS have been reported to activate NF-κB signalling [63]. The oxidative stress revealed in the current PDC-induced AKI may accounts for this elevation of renal NF-κB level. Previous studies identified the role of inflammatory transcription factors, specifically NF-kB family members in pathophysiology of AKI [64,65]. Because NF-κB plays a role in IL-18 induction and signalling [66,67], and has been identified in the promoter region of IL-18, this suggests an additional involvement of NF-κB in regulating IL-18 production [68,69]. Our results revealed a cross talk between NF-κB level and induction of renal IL-18 in PDC-induced AKI as evidenced by increased IL-18 level. Of note, this could provide a new insight into the mechanism underlying PDC-induced AKI.

Importantly, our results showed reduced levels of PDC-induced NF-κB in PDC-Lf-treated rats. Because these rats also exhibited reduced levels of PDC-induced IL-18, it is reasonable to speculate that Lf inhibits IL-18 expression by attenuating PDC-mediated NF-κB activation. In agreement, previous study reported the interference of Lf with lipopolysaccharide-induced NF-κB activation with reduction of cytokines production [53].

IGF-1 is a multifunctional hormone that has pleiotropic effects on cellular proliferation, apoptosis, hypertophy and differentiation [11]. When binds to its receptor (IGF-1R), IGF-I activates phosphatidylinositol-3 kinase (PI3k) / Akt signaling pathway [70]. Phosphorylated Akt is a natural stimulator of cell growth and proliferation, and a potent inhibitor of apoptosis. It functions through its downstream targets. FoxO1 transcription factor is a downstream target of Akt. Phosphorylation of FoxO1 by Akt inhibits its transcriptional functions and contributes to cell survival, growth and proliferation [71]. In the kidney, IGF-1 is expressed in a complicated manner and has profound effects on kidney growth, structure, and function [72,73]. IGF-1 dilates the resistance-regulating microvasculature, increases glomerular filtration rate, and promotes tubular phosphate and possibly sodium absorption [72,74]. It is known to protect against ischemic injury in rats [75,76]. On the other hand, IGF-1 could play a pathogenic role in hyperplasia of renal tubular epithelial cells and in the formation of renal cysts as it contributes to compensatory renal growth and may modestly contribute to progressive glomerular sclerosis [11–13,72].

The findings of the present study revealed elevated levels of renal IGF-1 and FoxO1 in rats with PDC-induced AKI. This could be considered as a compensatory mechanism of the kidney to protect against PDC-induced injury as it was found that IGF-I accelerates the recovery of renal function in AKI [77]. However, this elevation of renal content of IGF-1 may contribute to the progression of PDC-induced chronic renal injury [78]. These observed high levels of IGF-1 and FoxO1 may account for the tubular epithelial hyperplasia and apoptosis observed by the findings of the present PCNA, Bax and Caspase-3 immunohistochemical-staining. Compatible, previous studies reporting that overexpression of IGF-I in mice induces renal and glomerular hypertrophy [78]. In the present study, Lf markedly inhibited PDC-induced increase of renal contents of IGF-1 and FoxO1 and subsequently, decreased the tubular hypertrophy and apoptosis as shown by the current immunohistochemical findings. These results confirmed the protective effect of Lf against PDC-induced AKI. The antiproliferative effect of Lf has been reported in a previous study of our colleague [22], as well as in many other studies [79–82] that show inhibition of several growth factors by administration of Lf.

## Conclusion

The findings of this study revealed that oxidative stress and inflammation play major roles in PDC-induced AKI. Furthermore, the study demonstrated for the first time the involvement of IL-18 that could be one of the most important mediators of the renal tissue damage and tubular injury induced by PDC. It suggests the stimulation of Th2 response and IL-4-mediated inflammatory response as a mechanism of action for IL-18 in the current model.

Moreover, the study showed the involvement of IGF-1 which is known to play an important role in the pathogenic renal tissue hypertrophy, in PDC-AKI. It also records the upregulation of FoxO1 and suggests it as an important stimulator of the tubular epithelial hyperplasia and apoptosis observed in PDC-AKI.

Over and above, our results declared that the pretreatment of rats with Lf (200 and 300 mg/kg, p.o) produced marked protective effects against PDC-induced acute nephrotoxicity evidenced by biochemical results and proven by histopathological and immunohistochemical examinations. These findings revealed that this protective potential of Lf is possibly through its antioxidant, anti-inflammatory, and antiproliferative properties, and it suggested a role of IL-18 and IGF-1 inhibition.
